# Genome Sequences of Enteroinvasive Escherichia coli Sequence Type 6, 99, and 311 Strains Acquired in Asia Pacific

**DOI:** 10.1128/MRA.00944-19

**Published:** 2019-09-05

**Authors:** Rajat Dhakal, Qinning Wang, Peter Howard, Vitali Sintchenko

**Affiliations:** a Centre for Infectious Diseases and Microbiology-Public Health, Institute of Clinical Pathology and Medical Research-New South Wales Health Pathology, Westmead, New South Wales, Australia; b Marie Bashir Institute for Infectious Diseases and Biosecurity, Sydney Medical School, The University of Sydney, Sydney, New South Wales, Australia; University of Maryland School of Medicine

## Abstract

Laboratory diagnosis of enteroinvasive Escherichia coli (EIEC) remains difficult and limits the availability of EIEC genomes to the research community. We report the draft genome sequences of three EIEC strains which represent three distinct sequence types and serotypes circulating in Asia Pacific and causing enterocolitis in humans.

## ANNOUNCEMENT

Enteroinvasive Escherichia coli (EIEC) is closely related to Shigella species and causes enterocolitis syndrome, similar to shigellosis (1). However, the diversity and genomics of EIEC strains remain poorly understood due to the small number of available isolates and genomes and the difficulty of laboratory differentiation of EIEC from *Shigella* spp. Culture-independent direct testing (CIDT) on stool from patients with diarrhea has improved the recognition of EIEC-associated disease. The ironic reason for this has been the selection of the *ipaH* gene, which is present in *Shigella* species and EIEC, as the target for CIDT. This report contributes three new EIEC genomes representative of distinct subtypes and serotypes of EIEC causing gastrointestinal infections in humans in Asia Pacific.

Fecal isolates (*n* = 3) from New South Wales (NSW) (including one isolate from a patient with Pacific Islands travel history) were isolated in diagnostic laboratories throughout NSW and sent to the Centre for Infectious Diseases and Microbiology-Public Health (CIDM-PH), a public health microbiology reference laboratory in Sydney, Australia, for further characterization. Whenever required, isolates were grown in blood agar for 24 h at 37°C. We used our algorithm, which includes confirmation of the *ipaH* gene in isolates, identification of E. coli using the BD Phoenix automated identification and susceptibility testing system (Becton, Dickinson), and use of our in-house multiplex PCR assay (2), to identify and subtype EIEC. We then performed whole-genome sequencing (WGS) for in-depth characterization of the strains. Genomic DNA (gDNA) was extracted using the Geneaid Presto Mini gDNA bacteria kit. The extracted DNA was fragmented and tagged for multiplexing with Nextera XT DNA sample preparation kits (Illumina) and sequenced in house using the Illumina NextSeq 500 platform with 2 × 150-bp reads. Default parameters were used for the software used for analysis except where otherwise stated. Trimmomatic (v.0.36) (3) was used for cleaning fastq reads (LEADING: 3, TRAILING: 3, SLIDINGWINDOW: 4:20, MINLEN: 36). The Achtman 7-gene multilocus sequence typing (MLST) scheme in Enterobase and Nullarbor pipeline v.2.0.20181010 (4) containing ResFinder (v.3.1) and ABRicate (v.0.8) were used for subtyping and antibiotic resistome analysis. The Virulence Factors Database (VFDB), Snippy (v.4.3.5), and SKESA (v.2.3.0) were employed for virulome analysis, core genome analysis, and genome assembly, respectively.

The serotypes of these strains were identified as O121, O28, and O96, respectively. The genome sequencing statistics are presented in Table 1. The Achtman 7-gene MLST results showed that they belonged to sequence types 6, 311, and 99, respectively. Their subtypes from our multiplex PCR (2) were 3, 1, and 4 (common subtype). All strains contained the *ipgD* and *mxiA* genes associated with virulence. These two genes have been used to study various forms of pINV plasmids in EIEC strains (1). In addition, 19-0562-EIEC-0001 included a *gyrA* (D87N) mutation conferring *in vitro* resistance to fluoroquinolone antibiotics.

**TABLE 1:** Genome sequencing results

Sample	Coverage depth (fold)	Yr of isolation	Size (bp)	GC content (%)	No. of contigs	WGS-inferred serotype	Achtman 7-gene MLST	*N*_50_ (bp)	No. of reads
19-0562-EIEC-0001	122	2017	4,864,058	50.6	349	O121	6	28,116	3,645,936
18-0562-010	102	2018	4,797,511	50.5	385	O28	311	39,521	2,980,100
19-0562-EIEC-0006	148	2019	5,083,019	50.3	246	O96	99	100,937	4,284,180

### Data availability.

The genome sequences have been deposited in GenBank under the accession no. VMTQ00000000, VNKK00000000, and VNKL00000000. The versions described in this paper are the first versions, VMTQ01000000, VNKK01000000, and VNKL01000000, respectively. The SRA accession numbers for these samples are SRR9903352, SRR9903508, and SRR9903561, respectively.
